# Epsin2 promotes polarity establishment and meiotic division through activating Cdc42 in mouse oocyte

**DOI:** 10.18632/oncotarget.10815

**Published:** 2016-07-24

**Authors:** Ling Li, Longsen Han, Jiaqi Zhang, Xiaohui Liu, Rujun Ma, Xiaojing Hou, Juan Ge, Qiang Wang

**Affiliations:** ^1^ State Key Laboratory of Reproductive Medicine, Nanjing Medical University, Nanjing, China; ^2^ College of Animal Science and Technology, Nanjing Agricultural University, Nanjing, China; ^3^ Center of Reproductive Medicine, Nanjing Jinling Hospital, Nanjing University School of Medicine, Nanjing, China

**Keywords:** oocyte, meiosis, cytoskeleton, polarity, Epsin2, Pathology Section

## Abstract

Epsins are a conserved family of endocytic adaptors essential for diverse biological events. However, its role in oocytes remains completely unknown. Here, we report that specific depletion of Epsin2 in mouse oocytes significantly disrupts meiotic progression. Confocal microscopy reveals that Epsin2 knockdown results in the failure of actin cap formation and polar body extrusion during meiosis, indicative of the importance of Epsin2 in polarity establishment and cytokinesis. In addition, spindle defects and chromosome misalignment are readily observed in oocytes depleted of Epsin2. Moreover, we find that Epsin2 knockdown markedly decreases the activity of Cdc42 in oocytes and importantly, that the dominant-positive mutant of Cdc42 (Cdc42Q61L) is capable of partially rescuing the deficient phenotypes of Epsin2-knockdown oocytes. Together, our data identify Epsin2 as a novel player in regulating oocyte maturation, and demonstrate that Epsin2 promotes polarity establishment and meiotic division via activating Cdc42.

## INTRODUCTION

In both vertebrates and invertebrates, oocyte meiotic maturation is to generate a haploid gamete through two consecutive divisions, resulting in formation of a large oocyte and two small polar bodies [1]. To retain the most maternal stores within oocyte, meiotic divisions are highly asymmetric. By position the spindle from oocyte center to the cortex in meiosis I (MI), oocyte symmetry is broken and cortical polarity is established [2]. Cortex-located chromosomes induce a series of cortical remolding in oocytes, including disappearance of membrane microvilli, formation of a thick F-actin cap, and polarized accumulation of related molecules [3, 4]. This specialized cortical region defines the site of cytokinesis and polar body extrusion. It has been suggested that the signaling pathways controlling asymmetric cell divisions during meiosis is cued by chromatin and mediated by Ran GTPase gradient [5]. Cdc42 was identified as a critical molecular cascade initiated by Ran to establish polarity and control cytokinesis of oocytes [3, 6, 7]. Though the functions of Cdc42 in meiotic oocytes have been investigated, the underlying molecules involved in regulating Cdc42 activity remain to be discovered.

Epsins, as accessory factors, have been widely reported to participate in clathrin-mediated endocytosis. They consist of N-terminal homology (ENTH) domain, several ubiquitin interacting motif (UIMs) and peptide ligands that bind components of the endocytic machinery [8–10]. In addition to the putative roles in endocytosis, data from *yeast* showed that Epsins interact with Cdc42 GTPase-activating proteins (GAPs), which may regulate the levels of active Cdc42 [9]. On the other hand, the clathrin-mediated endocytosis proteins including endophilin, α-adaptin, and epsins, are also associated with the control of mitotic progression, possibly through affecting chromosome congression and spindle integrity [11–13].

Based on these findings, we propose that Epsins may act as the critical regulator of Cdc42 activity in oocyte meiosis, coordinating the polarity establishment and cytokinesis. However, to date, the role of Epsins in mammalian oocytes remains completely unknown. In the present study, by employing knockdown and overexpression analysis, we found that Epsin2 is predominately expressed in mouse oocyte, and specific depletion of Epsin2 disrupts the formation of actin cap and prevents polar body emission through affecting Cdc42 activity.

## RESULTS

### Epsins expression and cellular distribution in mouse oocytes

**Figure 1:**
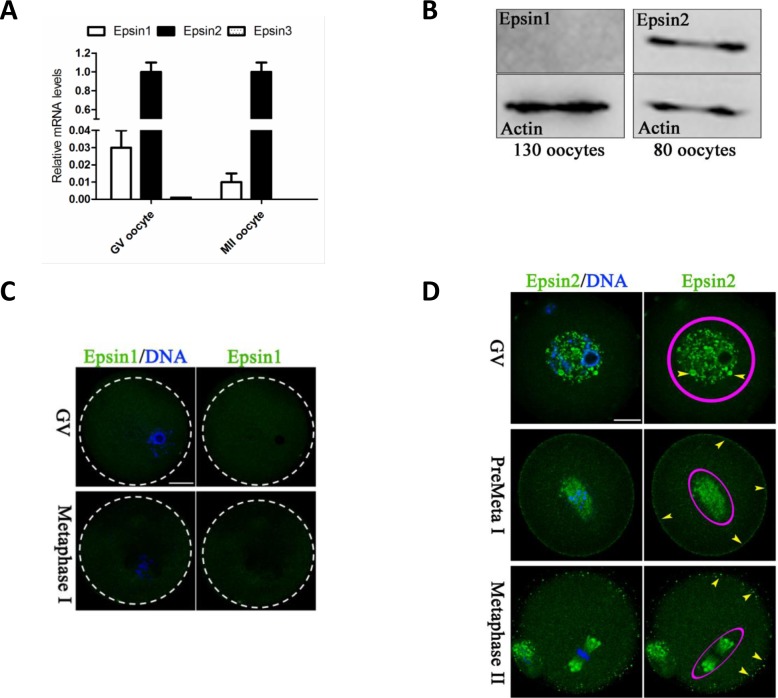
Epsins expression and cellular localization in mouse oocytes **A.** The relative mRNA levels of Epsin family were determined by qRT-PCR in GV and MII oocytes, with GAPDH as an internal control. **B.** Protein levels of Epsin1/2 in GV oocytes were determined by western blot, with actin as a loading control. **C.** GV and MI oocytes were immunolabeled with Epsin1 antibody (green), and counterstained with Hoechst 33342 (blue) for DNA. Dotted circles indicate the boundary of oocytes. **D.** Oocytes at GV, Pre-metaphase I, and metaphase II stage were immunolabeled with Epsin2 antibody (green), and counterstained with Hoechst 33342 (blue) for DNA. Epsin2 signals are denoted by circles and arrowheads indicate Epsin2-positive vesicles. Scale bar, 25μm.

We first evaluated the expression of different isoforms of Epsins in both GV and MII-stage oocytes. By performing quantitative real-time PCR, as shown in Figure 1A, we found that the level of Epsin3 mRNA was almost undetectable, with Epsin2 mRNA being most abundant and Epsin1 mRNA being less abundant. We further compared the protein level of Epsin1 and Epsin2 in GV oocytes. As shown in Figure 1B, Epsin2 band can be clearly detected in 80 oocytes pooled together, whereas Epsin1 band was hardly detected even with 130 oocytes. Similar results were observed by immunostaining analysis (Figure 1C-1D). These data suggested that Epsin2 is the predominant isoform of Epsin family in mouse oocyte.

Meanwhile, we examined the cellular localization of Epsin2 in mouse oocytes (Figure 1D). Epsin2-containing vesicles appear to be resided in the germinal vesicle (GV; arrowhead). As the oocytes enter into pre-metaphase stage (3h after GVBD), the signals become concentrated around chromosomes (circled area) and at the cortex (arrowheads). Likewise, at metaphase II (MII) stage, Epsin2 primarily localizes on the spindle region and its poles (circled area), and oocyte membrane (arrowheads). Such a dynamic and specific distribution pattern indicates that Epsin2 may have a function in regulation of oocyte meiosis.

### Perturbed meiotic progression in Epsin2-depleted oocytes

**Figure 2:**
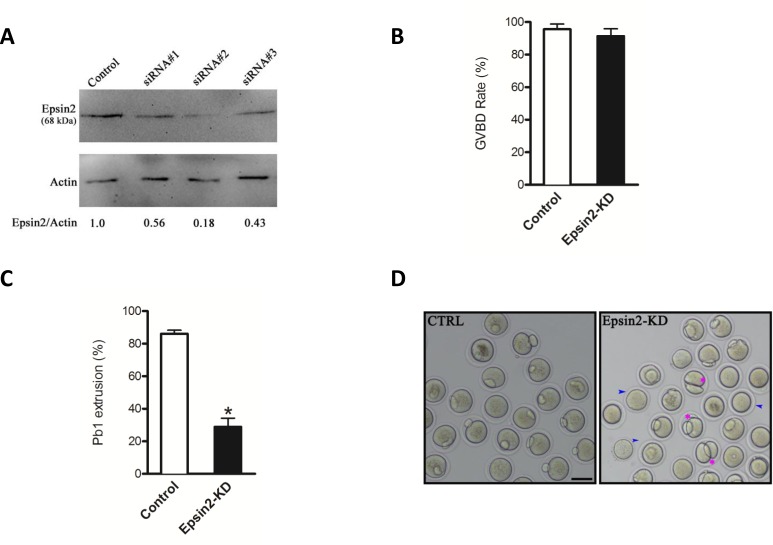
Disturbed meiotic progression in Epsin2-knockdown oocytes Fully grown oocytes injected with Epsin2 siRNAs were arrested in medium with milrinone for 20 hours, washed in milrinone-free medium, and then cutured *in vitro* for the experiments. Negative control siRNA was injected as control. **A.** Knockdown of endogenous Epsin2 protein after Epsin2 siRNA injection was verified by western blot. **B.**, **C.** Quantitative analysis of GVBD and Pb1 extrusion rate in control (*n* = 160) and Epsin2-KD (*n* = 143) oocytes. **D.** Representative images of oocytes from control and Epsin 2-KD groups. Blue arrowheads point to oocytes that fail to extrude polar body; pink asters denote oocytes with apparent symmetrical division. The graph shows the mean ± SD of the results obtained in three independent experiments. *p < 0.05 *vs*. controls. Scale bar, 50μm.

To investigate the function of Epsin2 during meiosis, we designed three siRNAs specifically targeted Epsin2. These Epsin2-siRNAs were separately microinjected into fully-grown oocytes to knock down the endogenous mRNA. Results showed that siRNA #2 led to the most dramatic reduction of Epsin2 protein in oocytes based on western blotting (Figure 2A), and therefore, this siRNA was used in the following experiments.

Our results showed that Epsin2 knockdown (Epsin2-KD) did not affect meiotic resumption, as the rates of germinal vesicle breakdown (GVBD) were similar after 3 hours *in vitro* culture (91.4± 4.5% *vs*. 95.6± 3.2% control, p> 0.05; Figure 2B). By contrast, the rate of first polar body (Pb1) emission was significantly decreased in Epsin2-KD oocytes when compared to controls (28.9 ± 5.3% vs. 86.1 ± 2.2% control, *p* < 0.05; Figure 2C), indicating that oocytes loss of Epsin2 failed to complete meiosis I and form Pb1 (Figure 2D, blue arrowheads). In a number of cases where a meiotic division appears to have been completed, a symmetrical “2-cell like” egg was frequently observed (around 15%; Figure 2D, pink asterisks). Together, these results suggest that Epsin2 is essential for oocyte maturation and meiotic divisions.

### Epsin2 knockdown induces defective spindle and misaligned chromosomes in oocytes

**Figure 3:**
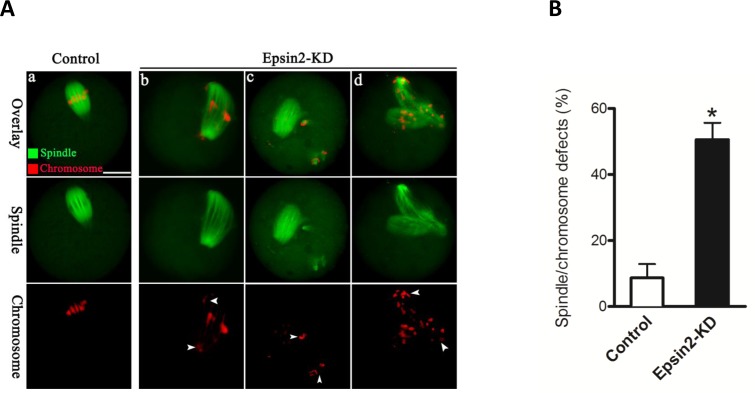
Epsin2 knockdown results in spindle defects and chromosome misalignment in oocyte meiosis **A.** Control and Epsin2-KD oocytes were stained with α-tubulin to visualize spindle (green) and counterstained with propidium iodide to visualize chromosomes (red): (a) A barrel-shape spindle and well-aligned chromosomes can be readily observed in control oocytes; (b-d) Chromosome congression failure indicated by arrowheads and disorganized spindles were frequenly detected in Epsin2-KD oocytes. **B.** Quantification of control (*n* = 182) and Epsin2-KD oocytes (*n* = 167) with spindle defects or chromosome misalignment. Data are expressed as mean percentage ± SD. *p < 0.05 *vs*. controls. Scale bar, 20μm.

We then asked whether depletion of Epsin2 affects the meiotic apparatus in oocytes. To address this question, oocytes from control and Epsin2-KD groups were fixed after 14 hours culture, and then immunolabeled with anti-tubulin antibody to visualize spindle and counterstained with propidium iodide (PI) for chromosomes. Confocal microscopy coupled with quantitative analysis revealed that oocytes depleted of Epsin2 displayed a high percentage of spindle/chromosome defects as compared with controls (51.6 ± 5.1% *vs*. 8.7 ± 4.2% control, *p* < 0.05; Figure 3B). These phenotypes mainly included diverse malformed spindles and multipolar spindles, as well as the displacement of one or several chromosomes from equator (Figure 3A, b-d, arrowheads). In contrast, most control oocytes presented a typically bipolar barrel-shaped spindle with well-aligned chromosomes at the equator (Figure 3A). These findings imply that the correct assembly of meiotic apparatus in mouse oocytes depends on Epsin2.

### Epsin2 is required for actin cap formation and polar body extrusion in oocytes

**Figure 4:**
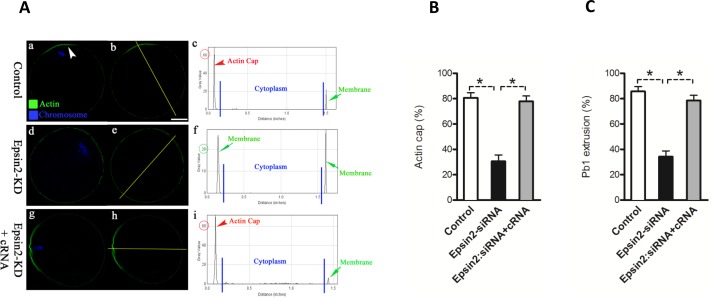
Epsin2 depletion disrupts actin cap formation and polar body extrusion in oocytes MII oocytes were labeled with phalloidin to visualize actin (green), and counterstained with Hoechst 33342 for chromosomes (blue). **A.** Representative confocal sections showing the actin distribution in control (a), Epsin2-KD (d), and Epsin2-siRNA+cRNA (g) oocytes. Arrowhead denotes the position of actin cap. Right graphs show the fluorescence intensity profiling of phalloidin in oocytes. Lines were drawn through the oocytes, and pixel intensities were quantified along the lines. **B.**, **C.** Quantitative analysis of control (*n* = 150), Epsin2-KD (*n* = 122), and Epsin2-siRNA+cRNA oocytes (*n* = 138) with intact actin cap and extruded polar body. Data are presented as mean ± SD from three independent experiments. *p < 0.05 *vs*. controls. Scale bar, 20μm.

Formation of cortical actin cap is one of the predominant features of oocyte polarization. This polarized region plays important role in polar body extrusion during meiosis [7]. To examine the effects of Epsin2 knockdown on actin polymerization, matured oocytes from control and Epsin2-KD groups were labeled with phalloidin and counterstained with Hoechst 33342. Confocal microscope revealed that polarized actin caps were readily observed in most normal oocytes with chromosomes approaching to the cortex (Figure 4Aa and 4B, arrowhead), further evidenced by the fluorescence plot profiling (Figure 4Ab-c). In striking contrast, the proportion of oocytes with obvious actin cap was significantly reduced when Epsin2 was abated in comparison to controls (30.5 ± 5.1% *vs*. 80.6 ± 4.0% control, *p* < 0.05; Figure 4A and 4B). In addition, the majority of Epsin2-KD oocytes showed the decreased intensity of phalloidin signals on membrane (Figure 4Ae-f).

To confirm the specificity of Epsin2 knockdown, we performed an mRNA rescue experiment, where the Epsin2 encoding cRNA contains mutations insensitive to the siRNA (see Materials and Methods). As shown in Figure 4Ag-I and 4B, Epsin2 overexpression could efficiently rescue the effects of siRNA knockdown, elevating the percentage of oocytes with intact actin cap (77.9 ± 4.2 *vs*. 30.5 ± 5.1% siRNA, p < 0.05; Figure 4Ag-I and 4B). Furthermore, the majority of rescued oocytes progressed to MII and extruded normal polar bodies as observed in controls (Figure 4C). In addition, Epsin2 overexpression did not significantly affect meiotic maturation in normal oocytes (data not shown). Collectively, these results suggest that Epsin2 is required for polarity establishment and cytokinesis during oocyte meiosis.

### Epsin2 promotes the polarity establishment in oocytes by activating Cdc42

**Figure 5:**
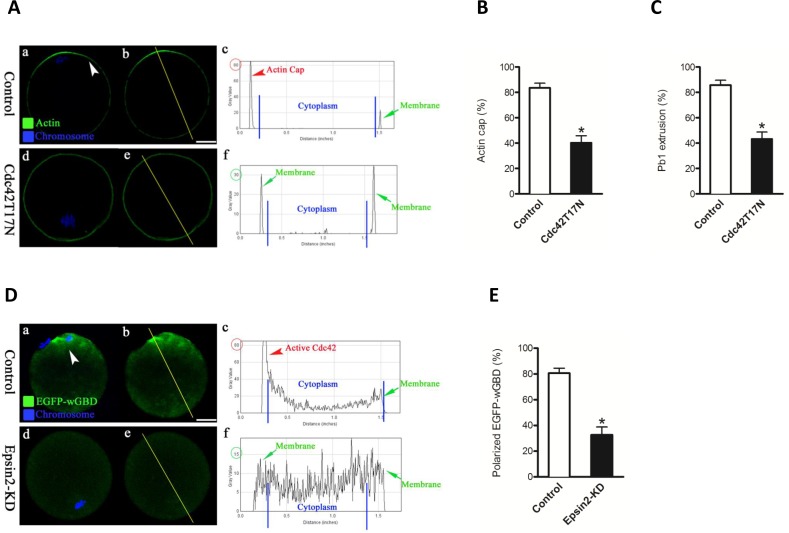
Loss of Episn2 results in the inactivation of Cdc42 in oocytes **A.** mRNAs encoding wild type (control) and dominant-negative mutant of Cdc42 (Cdc42T17N) were injected into fully grown oocytes to evaluate actin cap by Phalloidin (green) labeling. Chromosomes were stained with Hoechst 33342 (blue). **B.**, **C.** Quantitative analysis of control and Cdc42T17N oocytes with intact actin cap and extruded polar body. **D.** EGFP-wGBD mRNAs were injected into control and Epsin2-KD oocytes to trace the active Cdc42 (green) during meiosis, and chromosomes were stained with Hoechst 33342 (blue). Representative confocal sections are shown. Arrowhead denotes the position of the polarized EGFP-wGBD. Right graphs show the fluorescence intensity profiling of active Cdc42 in oocytes. Lines were drawn through the oocytes, and pixel intensities were quantified along the lines. **E.** Quantitative analysis of control (*n* = 80) and Epsin2-KD oocytes (*n* = 75) with polarized EGFP-wGBD signals. Data are presented as mean± SD from three independent experiments. *p < 0.05 *vs*. controls. Scale bar, 20μm.

Previous studies showed that Cdc42 is activated at the cortex overlying meiotic chromosomes and plays critical role in actin cap formation and polar body emission [3, 6]. Of note, ENTH domain of Epsin proteins has been found to interact with Cdc42-GAPs to potentially modulate the activity of Cdc42 in budding yeast [9]. These observations prompted us to hypothesize that Cdc42 might be a major downstream mediator of Epsin2 in establishing polarity in mouse oocytes. To address this question, we first confirmed the involvement of Cdc42 in the regulation of oocyte maturation using the GTPase-defective CDC42 mutants. Dominant-negative mutant of Cdc42 (Cdc42T17N) has a reduced affinity for nucleotides, and Cdc42Q61L has a defect in hydrolyzing GTP and is thus considered to be constitutively active [14]. Oocytes injected with wild type (control) and Cdc42T17N mRNA were collected for evaluating actin cap and polar body. Compared to controls, most oocytes expressing the Cdc42T17N mutant failed to form actin cap (Figure 5A-5B) and extrude polar body (Figure 5C), which is consistent with the published data [15].

**Figure 6:**
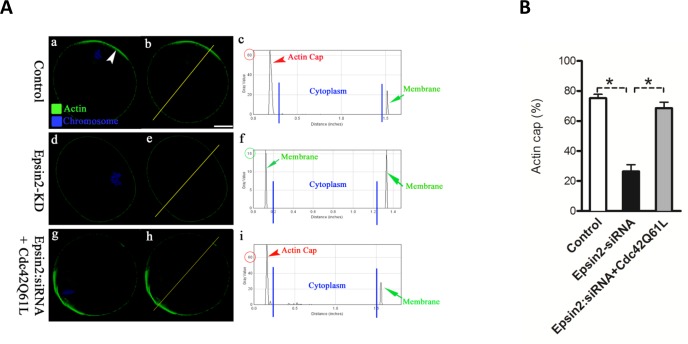
Dominant-positive mutant of Cdc42 (Cdc42Q61L) partially rescues the phenotypic defects in Epsin2-KD oocytes MII oocytes were labeled with phalloidin to visualize actin (green), and counterstained with Hoechst 33342 for chromosomes (blue). **A.** Representative confocal sections showing the actin distribution in control (a), Epsin2-KD (d), and Epsin2-siRNA+Cdc42Q61L (g) oocytes. Arrowhead denotes the position of actin cap. Right graphs show the fluorescence intensity profiling of phalloidin in oocytes. Lines were drawn through the oocytes, and pixel intensities were quantified along the lines. **B.** Quantitative analysis of control (*n* = 136), Epsin2-KD (*n* = 120), and Epsin2-siRNA+Cdc42Q61L (*n* = 122) oocytes with intact actin cap. Data are presented as mean ± SD from three independent experiments. *p < 0.05 *vs*. controls. Scale bar, 20μm.

Next, we determined whether Epsin2 knockdown affects the activity of Cdc42 in oocytes by employing the Cdc42-binding domain of N-WASP (wGBD) fused to enhanced green fluorescent protein (EGFP-wGBD). This Cdc42-GTP probe has been widely used to visualize active Cdc42 in different cell types [3, 16–18]. As expected, the polarized distribution of active Cdc42 was detected in normal metaphase oocyte (Figure 5Da-c; arrowhead). However, EGFP-wGBD signals were significantly decreased when Epsin2 was knocked down in oocytes (Figure 5Dd-f and Figure 5E), indicative of the Cdc42 inactivation. Therefore, we further examined whether a dominant-positive mutant, Cdc42Q61L, can rescue at least some of the phenotypic defects in Epsin2-KD oocytes. To this end, mRNA for Cdc42Q61L were microinjected into Epsin2-KD oocytes, and then matured oocytes were stained for checking actin cap. Our data showed that exogenous expression of Cdc42Q61L markedly increased the proportion of Epsin2-KD oocytes with typical actin cap, as shown in Figure 6A-6B. Taking together, these results demonstrated that Epsin2 promotes actin cap formation and meiotic division in oocytes through regulating the activity of Cdc42.

## DISCUSSION

Epsins are highly conserved clathrin-associated sorting proteins that are essential for endocytosis. Their functions are attributed to a membrane-active N-terminal ENTH domain, which binds phosphatidylinositol 4, 5-bisphosphate (PIP2) with high affinity, and multiple peptide motifs that mediate protein-protein interactions [19]. So far, in mammals, three members (Epsin1-3) of Epsin family have been identified. Epsin1 and 2 have been reported to be widely expressed in different tissues and cell types, while Epsin3 expression seems to be only induced by type I collagen in Wounded Epithelia [10, 20]. Here, by performing qRT-PCR and immunoblotting, we detected the expression of Epsin1 and 2 in mouse oocytes, with Epsin2 being most abundant and Epsin1/3 being least abundant (Figure 1), indicating that they may play differential roles. In this study, we primarily focus on the role of Epsin2 in oocyte meiosis.

Mammalian oocyte meiosis encompasses two rounds of highly asymmetric divisions to generate a functional haploid gamete and two small polar bodies [15]. Such asymmetry is ensured by the positioning of spindle in the periphery of large oocyte. In most mitotic cells, symmetry breaking is cued by extrinsic factors [21]. Nonetheless, the signals dictating polarity establishment are derived from chromosomes in meiotic oocytes [5]. In meiosis, bipolar spindle forms in the center of the oocytes and migrates to the nearest cortex. This movement is propelled by a cloud of dynamic actin filaments trailing behind the chromosomes/spindle. Chromosome approaching to the cortex induces cortical reorganization, which is marked by a local loss of microvilli, an accumulation of actin microfilaments under the plasma membrane (also termed actin cap), and the exclusion of cortical granules. This specialized region is may to generate a restriction domain for the assembly of contractile actin ring and cytokinesis in order to minimize the size of polar body [22]. In consistent with this notion, we found the failure to form actin cap and extrude polar body when Epsin2 was knockdown in oocytes (Figure 2). Thus, these findings suggest that Epsin2 is novel and important regulator of polarity establishment during oocyte meiosis.

Cdc42, one of the best characterized mammalian Rho GTPases, has multiple functions in controlling both microtubule and actin cytoskeleton [23]. Cdc42 acts as a molecular switch, cycling between an active GTP-bound state and inactive GDP-bound state. This cycle is regulated by its intrinsic GTPase activity and interaction with three protein families: guanine exchange factors (GEFs), guanine dissociation inhibitors (GDIs), and GTPase activating proteins (GAPs). Interestingly, ENTH domain of Epsins was demonstrated to interact with Cdc42-GAP and perhaps affect Cdc42 activity in budding yeast [9]. Previous work has shown that Cdc42 is activated in cortical region overlying meiotic chromosomes, which depends on Ran-GTP gradient. Furthermore, expression of Cdc42T17N significantly inhibited the formation of cortical actin cap and meiotic division in mouse oocytes (Figure 5) [24]. It is worth noting that these phenotypes are very similar to what we observed in Epsin2-KD oocytes. More importantly, polarized accumulation of Cdc42-GTP was lost in Epsin2-depleted oocytes (Figure 5). Cdc42Q61L could partially rescue the phenotypic defects of Epsin2-KD oocytes (Figure 6). On the basis of these data, we conclude that Epsin2 is involved in the establishment of oocyte polarity through regulating the activity of Cdc42 during meiosis.

In addition to the function in cell polarity, we also observed the spindle defects and chromosome congression failure in Epsin2-KD oocytes (Figure 3). In line with this phenotype, Cdc42 was found to be able to modulate microtubule attachment to kinetochores via its effector mDia3, and expression of Cdc42T17N induced the scattered chromosomes in somatic cells [25]. Our ongoing research is trying to explore the potential mechanism through which Epsin2 controls meiotic apparatus in mammalian oocytes.

In summary, cortical polarity is essential for oocyte maturation. Failure to establish polarity impairs oocyte competence, contributing to abnormal embryonic development and birth defects [26]. Our data indicate a role for Epsin2 in actin cap formation and cytokinesis in oocytes, which opens a new area for understanding mechanisms controlling germ cell development as well as assessing oocyte quality.

## MATERIALS AND METHODS

All materials were purchased from Sigma unless otherwise stated. ICR mice were used in this study. All experiments were approved by the Animal Care and Use Committee of Nanjing Medical University and were performed in accordance with institutional guidelines.

### Antibodies

Antibodies were obtained from the following commercial sources: mouse monoclonal anti-α-tubulin-FITC antibody (Sigma, Cat# T6074); goat polyclonal anti-Epsin1 antibody (Santa Cruz, Cat# sc-8673); mouse monoclonal anti-Epsin2 antibody (Santa Cruz, Cat# sc-376788); FITC-conjugated Phalloidin (Sigma, Cat# P5282); FITC-conjugated goat anti-mouse IgG (Thermo Fisher Scientific, Cat# 31569); FITC-conjugated donkey anti-goat IgG (Jackson Immuno Research Laboratory, Cat# 705-095-147).

### Oocyte collection and culture

Female ICR mice (6-8 weeks old) were sacrificed after superovulation by injecting 5IU pregnant mare serum gonadotropin (PMSG; Sigma-Aldrich). Cumulus-enclosed oocytes were collected, and then denuded by repeatedly mouth-pipetting. For *in vitro* maturation, fully-grown GV oocytes were cultured in M16 medium under mineral oil at 37°C in a 5% CO_2_ incubator.

### siRNA, plasmid construction and mRNA synthesis

siRNAs for mouse Epsin2 (siRNA#1, 5′-GTGATGACCTCAGATTGCATT-3′; siRNA#2, 5′-CTGTCCCTAAGAACTCAGATT-3′; siRNA#3, 5′-GTAATTTCAACGGTACAGTTT-3′) were purchased from GenePharma (Shanghai, China) and then diluted with water to give a stock concentration of 1 mM.

Plasmid encoding the EGFP-wGBD in pCS2+ vector was kindly provided by Dr. Zhen-Bo Wang (Chinese Academy of Sciences, Beijing, China). Dominant-negative mutant (Cdc42T17N) and dominant-positive mutant (Cdc42Q61L) (from Dr. Ping Zheng, Chinese Academy of Sciences, Kunming, China) were subcloned in pcDNA3.1. The full length of Epsin2 cDNA was cloned by PCR using the primers: F1 5′-GGGGGCCGGCCATGACAACTTCATCTATCA-3′ and R1 5′-GGG GGCGCGCCCTAGAGAAGGAAAGGGTTT-3′. To obtain the rescue construct for siRNA#2, six wobble codon mutations in the central siRNA#2-binding region were introduced by overlapping PCR (5-CT GTC CCT AAG AAC TCAGA -3 to 5 - CA GTA CCA AAA AAT TCG GA-3;underlining indicates the nucleotides mutated).

For *in vitro* mRNA synthesis, expression constructs were linearized by ScaI (for Cdc42T17N and Cdc42Q61L), SalI (for EGFP-WGBD), and NotI (for Epsin2). Capped mRNAs were synthesized with T7 (for pcDNA3.1 vector) or SP6 (for pCS2+ vecter) polymerase (mMessagem Machine Kit, Ambion). mRNAs were purified by RNeasy Micro Kit (Qiagen, Germany) according to the manufacturer's instruction and stored at −80°C.

### Knockdown and overexpression analysis

siRNA (for knockdown experiment) and/or mRNA (for overexpression experiment) were injected into fully-grown GV oocytes by using a Narishige (Tokyo, Japan) microinjector. In order to facilitate the siRNA-mediated knockdown or mRNA translation, oocytes were arrested at GV stage in M2 medium containing 2.5 μM milrinone for 20-24 hours, and then cultured in milrinone-free M2 medium for further experiments.

### Quantitative real-time PCR

Total RNA was isolated from 40 oocytes using a PicoPure RNA Isolation Kit (Thermo Fisher Scientific). Reverse transcription was performed with random primers. cDNA was quantified by qRT-PCR using an ABI Stepone Plus Real-time PCR system (Applied Biosystems, CA, USA). The fold change in gene expression was normalized with glyceraldehydes-3-phosphate dehydrogenase (GAPDH) using the comparative ΔΔCt method. Primer sequences are listed below:

Epsin1-F: 5′-CTGGGGCATTCGACATGAGT-3′

Epsin1-R: 5′-CCACAAGGGCTGCATTAGGG-3′

Epsin2-R: 5′-CAGCCAGCCCAACCTTTCTAC-3′

Epsin2-F: 5′-GCTGTCTGCACGCTTTCTGA-3′

Epsin3-F: 5′-ATTGTTCACAACTACTCTGAGGC-3′

Epsin3-R: 5′-GCCACCGTGTTGAAGGTCA-3′

### Western blotting

Oocytes were lysed in SDS sample buffer with protease inhibitor and heated for 5 min at 100°C. The denatured proteins were separated by gel electrophoresis and electrically transferred to PVDF membrane. Blocking of non-specific binding is achieved by placing the membrane in a dilute solution of 5% low-fat dry milk for 1 h, and then primary antibodies were incubated overnight at 4C. After washing three times in PBST to remove unbound primary antibody, the membranes were incubated with secondary antibodies for 1 hour at room temperature. After three washes, bands were visualized by an ECL Plus Western Blotting Detection System (GE Healthcare, Piscataway, NJ, USA). Finally, the membranes were washed in stripping buffer and reblotted with anti-actin antibody (1:5,000) for loading control.

### Immunofluorescence and confocal microscopy

For staining of Epsin1/2 and α-tubulin, oocytes were fixed in 4% paraformaldehyde for 30 min and permeabilized with 0.5%Triton X-100 for 20 min. After 1h incubation in 1% BSA in PBS, samples were incubated overnight at 4°C with primary antibodies. After three washes in PBST, oocytes were labeled with FITC- or Cy5-conjugated secondary antibody for 1 hour at room temperature. Chromosome was stained with propidium iodide (red) or Hoechst 33342 (blue) for 10 min. After brief washes, oocytes were mounted on glass slides in a drop of anti-fade medium (Vectashield, CA, USA), and then examined with a Laser Scanning Confocal Microscope (LSM 710; Zeiss, Oberkochen, Germany).

For staining of F-actin, oocytes were fixed in 3.7% paraformaldehyde for 5 min, blocked for 1 hour at room temperature, and incubated with Phalloidin-FITC for 1 hour. Following three washes, oocytes were stained with Hoechst 33342 for nuclear. To detect active Cdc42, oocytes injected with EGFP-wGBD probe were cultured for 12 hours and attached to dish for imaging. Samples were examined by a Laser Scanning Confocal Microscope. ROI measurement was applied to quantify actin fluorescence of each oocyte images. Fluorescence intensity was randomly measured by plot profiling using ImageJ software (NIH, USA).

### Statistical analysis

Data are presented as means ± SD, unless otherwise stated. Statistical comparisons were made with Student's test and ANOVA when appropriate. *P* < 0.05 was considered to be significant.
